# Transmission Dynamics of Crimean–Congo Haemorrhagic Fever Virus (CCHFV): Evidence of Circulation in Humans, Livestock, and Rodents in Diverse Ecologies in Kenya

**DOI:** 10.3390/v15091891

**Published:** 2023-09-07

**Authors:** Dorcus C. A. Omoga, David P. Tchouassi, Marietjie Venter, Edwin O. Ogola, Josephine Osalla, Anne Kopp, Inga Slothouwer, Baldwyn Torto, Sandra Junglen, Rosemary Sang

**Affiliations:** 1 International Centre of Insect Physiology and Ecology, Nairobi P.O. Box 30772-00100, Kenya; dorcusomoga@gmail.com (D.C.A.O.); dtchouassi@icipe.org (D.P.T.); eogola@icipe.org (E.O.O.); josalla@icipe.org (J.O.); btorto@icipe.org (B.T.); 2 Zoonotic Arbo and Respiratory Virus Research Program, Centre for Viral Zoonoses, Department of Medical Virology, Faculty of Health, University of Pretoria, Private Bag X 323, Gezina 0031, South Africa; marietjie.venter@up.ac.za; 3 Institute of Virology, Charité Universitätsmedizin Berlin, Corporate Member of Free University Berlin, Humboldt-University Berlin, and Berlin Institute of Health, Chariteplatz 1, 10117 Berlin, Germany; anne.kopp@charite.de (A.K.); inga.slothouwer@charite.de (I.S.)

**Keywords:** Crimean–Congo haemorrhagic fever, serological surveillance, peridomestic rodents, livestock, febrile patients, Kenya

## Abstract

Crimean–Congo haemorrhagic fever virus (CCHFV) is the causative agent of CCHF, a fatal viral haemorrhagic fever disease in humans. The maintenance of CCHFV in the ecosystem remains poorly understood. Certain tick species are considered as vectors and reservoirs of the virus. Diverse animals are suspected as amplifiers, with only scarce knowledge regarding rodents in virus epidemiology. In this study, serum samples from febrile patients, asymptomatic livestock (cattle, donkeys, sheep, and goats), and peridomestic rodents from Baringo (Marigat) and Kajiado (Nguruman) counties within the Kenyan Rift Valley were screened for acute CCHFV infection by RT-PCR and for CCHFV exposure by ELISA. RT-PCR was performed on all livestock samples in pools (5–7/pool by species and site) and in humans and rodents individually. CCHFV seropositivity was significantly higher in livestock (11.9%, 113/951) compared to rodents (6.5%, 6/93) and humans (5.9%, 29/493) (*p* = 0.001). Among the livestock, seropositivity was the highest in donkeys (31.4%, 16/51), followed by cattle (14.1%, 44/310), sheep (9.8%, 29/295) and goats (8.1%, 24/295). The presence of IgM antibodies against CCHFV was found in febrile patients suggesting acute or recent infection. CCHFV RNA was detected in four pooled sera samples from sheep (1.4%, 4/280) and four rodent tissues (0.83%, 4/480) showing up to 99% pairwise nucleotide identities among each other. Phylogenetic analyses of partial S segment sequences generated from these samples revealed a close relationship of 96–98% nucleotide identity to strains in the CCHFV Africa 3 lineage. The findings of this study suggest active unnoticed circulation of CCHFV in the study area and the involvement of livestock, rodents, and humans in the circulation of CCHFV in Kenya. The detection of CCHF viral RNA and antibodies against CCHFV in rodents suggests that they may participate in the viral transmission cycle.

## 1. Introduction

Crimean–Congo haemorrhagic fever virus (CCHFV) is a tick-borne zoonotic virus in the species *Crimean–Congo haemorrhagic fever orthonairovirus*, genus *Orthonairovirus*, family *Nairoviridae* in the order Bunyavirales [1,2]. CCHFV has a tripartite, linear, single-stranded, negative-sense RNA genome. The three segments small (S), medium (M), and large (L) encode the nucleocapsid (N) and the NSs non-structural proteins, the two glycoproteins Gn and Gc and a non-structural protein NSm, and the RNA-dependent RNA-polymerase, respectively [3,4,5,6]. The virus causes haemorrhage, myalgia, and fever in humans and the disease was first described in Crimea in 1944–1945 [7]. It was later shown that the same virus was responsible for an illness identified in 1956 in the Democratic Republic of Congo and therefore was named the Crimean–Congo Haemorrhagic Fever virus [8,9,10]. The case fatality rate in CCHFV outbreaks is 10–40% according to the World Health Organisation (WHO) report [11]. CCHFV is classified into seven genetic lineages (Africa 1, 2, and 3, Asia 1 and 2, and Europe 1 and 2) that cause severe disease in humans, except the Europe 2 lineage [12].

Numerous wild and domestic animals, such as cattle, goats, sheep, ostriches, and hares, can become infected with CCHFV but do not show clinical symptoms of the disease [13,14,15,16,17]. These vertebrates act as amplification hosts. Although the viraemic phase is short in a majority of vertebrate hosts lasting 2–7 days [18,19], these hosts amplify the virus and support the virus spread from one tick to another [20,21,22]. Belobo et al. (2021) reported an overall worldwide CCHFV seroprevalence of 12.0% in animal species, whereas Spengler et al. (2016) stated seroprevalence of 19.3%, 21.7%, 28.1%, and 23.9% in cattle, donkey, goats, and sheep, respectively [23,24]. In Africa, much higher seroprevalence rates have been reported in livestock species, e.g., Uganda (36.5%), Zimbabwe (37%), Mauritania (67%), South Africa (74.2%) and Senegal (32.5%) [25,26,27,28]. However, few studies detected the CCHFV nucleic acids in vertebrates, most probably due to the short viraemic phase [29,30,31,32,33].

Rodents and shrews are widely distributed in peridomestic habitats and contact with human populations often occurs with a potential risk of disease transmission [34]. The rodent species commonly found in human settlements, e.g., *Rattus rattus*, *Mastomys natalensis*, *Crocidura* spp., and house mice (*Mus* spp.), are known to vector medically important hantaviruses and arenaviruses but also arboviruses like Wesselsbron (WSL) and Usutu (USUV) viruses [34,35,36]. However, the role of rodents in CCHFV transmission is not well understood, despite the fact that they can become infected through parasitized infected immature tick species, which in turn can infect/transmit the virus to humans who live in close proximity [37]. Whether CCHFV could be transmitted from rodent to human through other transmission pathways like urine, faces, rodent meat consumption, and other body fluids/secretions like rodent-borne viruses requires further investigations that will decipher their possible role as amplification/reservoir hosts in the CCHFV transmission cycle. Various studies have reported the presence of CCHFV antibodies in the mouse species *Apodemus sylvaticus*, brown rat *Rattus norvegicus* (Pakistan), Bushveld gerbil (*Gerbilliscus leucogaster*), *Aethomys namaquensis*, *Rhabdomys pumilio*, and *Mastomys* spp. (South Africa/Zimbabwe), *Rattus rattus* (Pakistan), *Arvicanthis niloticus* and *Mastomys erythroleucus* (Mauritania), and unidentified rodents in Iraq and Iran, among others [26,37,38,39,40]. Thus, rodents could play an important role in the transmission of CCHFV.

CCHFV is transmitted by ticks that serve as vectors and reservoirs and play an important role in CCHFV maintenance [41]. *Hyalomma* ticks of the family *Ixodidae* are the main vector and reservoir of the virus [9,10,26,42]. CCHFV has also been detected in other tick species of the genera *Rhipicephalus*, *Amblyomma*, and *Dermacantor*, although their role in virus maintenance and transmission is unclear [7,9,18]. Ticks are considered reservoirs as they support life-long and transstadial infections in contrast to short-term viremia observed in vertebrates [13,23,24,41]. Furthermore, non-systemic transmission of CCHFV can occur between ticks through co-feeding on a non-viraemic host as well as through venereal routes during mating [9,37,43,44,45,46].

Besides infection via the bite of a CCHFV-infected tick, humans can also become infected through contact with blood, secretions, organs, or other body fluids of infected persons and livestock during slaughter. Consequently, this makes people who are in close contact with animals like pastoralists, veterinarians, and abattoir workers more prone to CCHFV infections.

CCHFV is widely distributed and endemic in some parts of Africa, Europe, the Middle East, and Asia. The broad geographic spread is believed to be influenced by several factors including the wide distribution of the primary vector (*Hyalomma* ticks), the prolonged maintenance of the virus in ticks through horizontal and vertical transmission, the extended feeding times associated with ticks as well as the long-distance movement of livestock [9,10,47].

In Kenya, CCHFV was first detected in *Rh. pulchellus* in 1970 from dying sheep in the Kabete veterinary laboratories [7]. The first documented case of acute human infection with CCHFV was in the year 2000 from a farmer in western Kenya [47]. Subsequent studies have detected the virus in diverse tick species infesting livestock and antibodies against CCHFV were found in humans through serosurveys [15,16,30,31,48]. For instance, in Northeastern Kenya, the virus was detected in *Hy. rufipes* and *Hy. truncatum* infesting cattle and camels, and recently in *Rh. decoloratus* in western Kenya [30,31]. A recent study in Baringo and Kajiado counties confirmed livestock infestation with *Rhipicephalus* spp. and *Hyalomma* spp. ticks [49], which represent potential risk factors for CCHFV circulation. Furthermore, several serosurveys have revealed CCHFV seroprevalence rates of 14–35% in febrile patients from different geographical areas in Kenya [15,16,50]. Only a few studies have monitored CCHFV infection rates in livestock and wildlife in Kenya [29,32]. Much less investigated is the role of rodents in CCHFV transmission.

In this study, a one-health surveillance approach of CCHFV detection among a network of vertebrate hosts including humans was implemented. The study was conducted in two pastoralist-dominated areas, Kajiado and Baringo counties, in the Kenyan Rift Valley to help understand CCHFV transmission and circulation in these ecosystems. Specifically, evidence of CCHFV circulation and exposure in inapparent asymptomatic livestock, including cattle, goats, donkeys, sheep, and peridomestic rodents, was assessed. Additionally, syndromic surveillance of CCHFV in patients with febrile illness was conducted to determine the possible contribution of the virus to fevers of unknown origin.

## 2. Materials and Methods

### 2.1. Ethical Approval

The study was approved by the Kenya Medical Research Institute’s Scientific and Ethics Review Unit (SERU), (SERU No.3312) after gaining approval by the animal care and use committee. Additional approval for the study was accorded by the University of Pretoria, Faculty of Health Science’s Research Ethics Committee (Ethics Reference No. 568/2020).

Sampling of livestock (cattle, goats, and sheep) was conducted by a team including a Kenya Veterinary Board (KVB)-registered veterinarian or/an animal health technician after obtaining written consent from the respective county governments and verbal consent from local farmers. Additionally, informed written consent was obtained from all febrile patients sampled.

### 2.2. Study Sites

**Figure 1:**
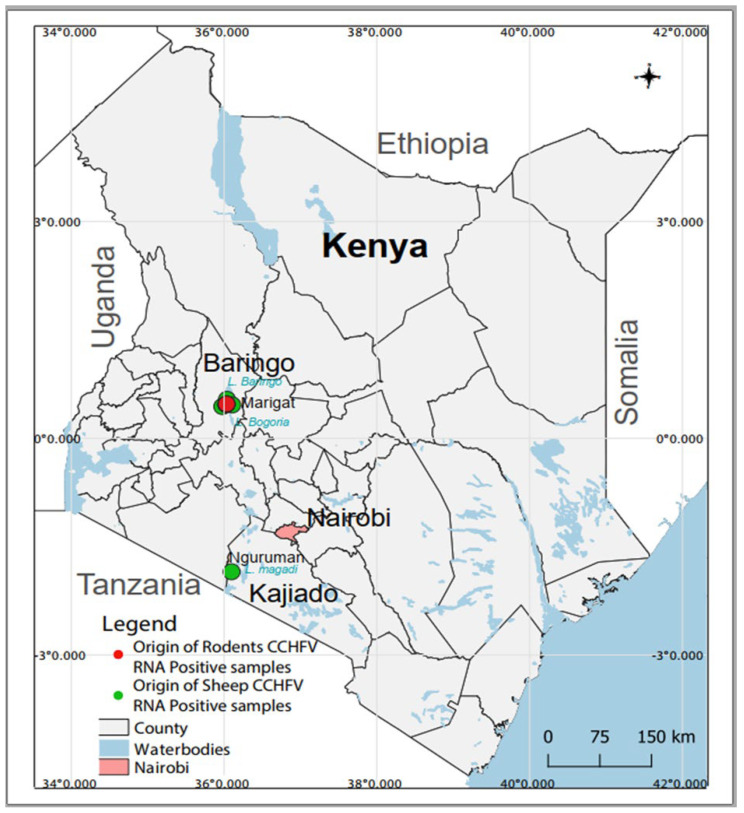
Map of Kenya showing sampling sites and the origin of CCHFV RNA-positive samples. The map was created in the open source GIS software, QGIS 3.22 using GPS co-ordinates and shape files derived from Natural Earth (http://www.naturalearthdata.com/, a free GIS data source accessed on 20 April 2023) and Africa Open data (https://africaopendata.org/dataset/kenya-counties-shapefile, license Creative Commons, accessed on 20 April 2023).

The survey was conducted in different locations within Baringo (0.4695° N, 35.9833° E) and Kajiado (1.7617° S, 36.0255° E) counties from January 2020 to December 2021 as part of a larger project that aimed at understanding the transmission dynamics of arboviruses [51,52]. Both field sites are located in the Kenyan Rift Valley and are semi-arid ecologies mainly inhabited by nomadic pastoralist communities and a history of arbovirus circulation [16,49,50,53,54,55] (Figure 1).

### 2.3. Human Sampling

Human sampling was conducted at Marigat Subcounty Hospital (Baringo County) and Entasopia Health Centre (Kajiado County). Patients (male and female) ≥5 years presenting with a clinical case definition of acute febrile illness characterized by fever (body temperature ≥ 38 °C) and at least one of the following clinical manifestations: cough, joint pain, headache, chills, general body malaise, and any signs of bleeding and neurological abnormalities were formally recruited for the study after obtaining their written consent and, in the case of children, consent from guardians. Clinical demographics and risk factor data were collected from the patients using a questionnaire and a data sheet.

A total of 5 mL of blood from each participant was collected into BD Vacutainer^®^ serum tubes (Becton, Dickinson and Company, Franklin Lakes, NJ, USA) with a clot activator by a trained and licensed phlebotomist. The serum was separated by centrifuging at 1500 rpm for 10 min using an Eppendorf™ 5702 Series Centrifuge. Samples were stored in liquid nitrogen at the health facilities until transportation on dry ice to the Emerging Infectious Disease (EID) Laboratory at *icipe*, Nairobi, where they were stored at −80 °C until further analysis.

### 2.4. Livestock Sampling

A random sampling of domesticated livestock (cattle, sheep, goats, and donkeys) aged 1–3 years was conducted twice a year after the short and long rains by a registered veterinarian and/or an animal health technician after obtaining verbal consent from the farmers. Animal characteristics like age and sex as well as information on vaccination history, clinical signs, and location of sampling were recorded in a data capture form. Animals were randomly selected and whole blood from the jugular vein was collected using a 10 mL BD Vacutainer^®^ with EDTA and one pre-coated with serum activator for serum. Samples were transported on dry ice to the EID Laboratory at *icipe*, Nairobi, and stored at −80 °C until further analysis.

### 2.5. Sampling of Peridomestic Rodents

Rodents were trapped using the LFAHD Folding Live Capture Rodent/Rat/Mouse traps (https://www.shermantraps.com/animal-traps/). Two to four traps were placed inside houses and their surroundings within the study sites (Baringo and Kajiado counties) according to the National Museum of Kenya (NMK) guidelines (unpublished). The traps were baited with a mixture of locally available peanut butter and white oats, opened at dusk, checked every morning, and left closed during the day. Trapped specimens were placed in a handling bag, weighed, had their sex and age recorded in a data capture form, and identified to the genus or species based on morphological and geographical criteria according to the Kingdon Guide to African Mammals [56] and East African Mammals [57]. Species were further confirmed by molecular analyses [58,59].

The rodents were euthanatized by cervical dislocation, and tissues (kidney, spleen, lung, heart, and liver samples) and blood were collected using cryovials and BD Vacutainer^®^ tubes with serum activator, respectively. Serum was separated through centrifugation at 3000 rpm for 5 min. Samples were stored in liquid nitrogen until transportation to the EID Laboratory at *icipe*, Nairobi, where they were stored at −80˚C.

### 2.6. CCHFV IgG and IgM ELISA

The presence of CCHFV IgG antibodies in febrile patients was detected by ELISA according to the manufacturer’s instruction (VectoCrimean-CHF-IgG kit; Vector-Best, Russia, https://vector-best.ru). Positive samples were further tested for the presence of IgM antibodies by ELISA according to the manufacturer’s instruction (VectoCrimean-CHF-IgM ELISA kit; Vector-Best, Russia, https://vector-best.ru). Livestock and rodent serum samples were screened for CCHFV IgG antibodies by ELISA using the ID Screen^®^ CCHF Double Antigen Multi-species ELISA kit (ID Vet, France, https://www.id-vet.com) according to the manufacturer’s instructions. Optical density (OD) values were determined using a BioTek ELX800 Microplate reader at 450 nm.

### 2.7. Statistical Data Analysis

Demographic data and the presence of CCHFV IgM and IgG antibodies expressed as positive or negative were analyzed using R version 4.2.0. Descriptive and univariable analyses were performed to understand how variables like age, gender, location as well as occupation and contact with animals could be linked to CCHFV exposure in humans. A comparison between the two study sites and different hosts was conducted using the chi-square test. The Agresti–Coull method was used to estimate the 95% confidence intervals (CIs). All tests were performed at a 5% significance level. Multiple logistic regression analysis based on likelihood ratio (LR) testing at a 5% level model reduction was performed to understand risk factors associated with CCHFV seropositivity.

### 2.8. RNA Extraction, PCR Screening, and Sequencing

Viral RNA was extracted from individual human serum samples, homogenized rodent tissues, and pooled livestock sera (consisting of 5–7 individual samples according to site and species) using the QIAamp Viral RNA Minikit (QIAGEN, Hilden Germany) according to the manufacturer’s protocol. Invitrogen SuperScript™ III Reverse Transcriptase (Thermo Fisher Scientific Inc., Waltham, MA, USA) was used for cDNA synthesis and samples were screened by RT- PCR using the primers CCHFV F2 (5′-TGGACACCTTCACAAACTC-3′) and CCHFV R3 (5′-GACAAATTCCCTGCACCA-3′) amplifying a fragment of the nucleocapsid gene of 536 bp [60]. PCR products were analyzed using a 2% agarose gel stained with Invitrogen UltraPure™ Ethidium Bromide (Thermo Fisher Scientific Inc., USA). Amplicons were purified using the Applied Biosystems™ ExoSAP-IT™ PCR Product Cleanup Reagent (Thermo Fisher Scientific Inc., USA) according to the manufacturer’s instructions and sequenced in both directions at Macrogen, Europe B.V. Sequences were cleaned in Geneious Prime software (Version 2022.1.1) (https://www.geneious.com) and queried in the GenBank-NCBI database using the Basic Local Alignment Search Tool (BLAST) [61]. Sequencing of entire CCHFV genomes was attempted using the Illumina MiSeq platform as described previously [62].

### 2.9. Phylogenetic Analysis

CCHFV S segment sequences were downloaded from the GenBank NCBI database and a multiple sequence alignment with the Nairobi sheep disease virus (NSDV_KM464724) as an outgroup was performed using MAFFT [63]. A maximum likelihood phylogenetic analysis using PhyML [64] and the general time reversible (GTR) model and applying 1000 bootstrap replicates was inferred.

## 3. Results

### 3.1. CCHFV Serology

#### 3.1.1. CCHFV Antibody Detection in Humans

A total of 493 human samples were analyzed—323 (65.5%) from Marigat and 170 (34.5%) from Nguruman, with a higher proportion of females (295/493, 59.8%) than males (198/493, 40.2%) (Table 1). In total, 5.9% (29/493) showed the presence of IgG antibodies against CCHFV with a higher proportion of positive samples originating from Marigat (22/29, 75.9%) than from Nguruman (7/29, 24.1%), albeit the difference was not significant (*p* = 0.23) (Table 1). The seropositivity proportion did not differ by sex or age category (Table 1). Occupation was a poor predictor of CCHFV exposure in humans based on IgG antibodies. Humans in contact with domesticated animals were 1.4 times more likely to be positive for CCHFV antibodies, but this did not pose a risk of CCHFV infection (*p* = 0.62). Location, age group, gender, and occupation did not affect CCHFV seropositivity (Table 1 and Table 2). Additionally, 9 of the 29 IgG-positive samples showed the presence of IgM antibodies (9/29, 31%), supportive of an acute or recent infection with CCHFV. No significant difference in age, gender, and location was found for IgM-positive cases (Table 2). There was a significant positive correlation between the presence of CCHFV IgM antibodies and retro-orbital pain symptoms (*p* = 0.042) (Table 3).

#### 3.1.2. CCHFV Antibody Detection in Livestock

In total, 951 livestock serum samples comprising cattle (*n* = 310), goats (*n* = 295), sheep (*n* = 295), and donkeys (*n* = 51) were screened for the presence of IgG antibodies against CCHFV. A larger proportion of the samples were from Marigat (*n* = 551) than from Nguruman (*n* = 400) (Table 4). An overall CCHFV seropositivity of 11.9% (113/951; 95% CI, 10–14.1; Table 4) was found. Seropositivity did not differ between Marigat and Nguruman (12.7% vs. 10.8%; 95% CI, 1.2 (0.80–1.80) *p* = 0.36) and not between females and males (11.6% vs. 12.7%; 95% CI, 1.1 (0.72–1.70), *p* = 0.63) (Table 5) but it increased with age. However, livestock in Marigat were 1.2 times more likely to be seropositive for CCHFV compared to those in Nguruman. Within each species, the odds of testing positive for CCHFV antibodies were similar in males and females (OR = 1.1, df 1, *p* = 0.24).

**Figure 2:**
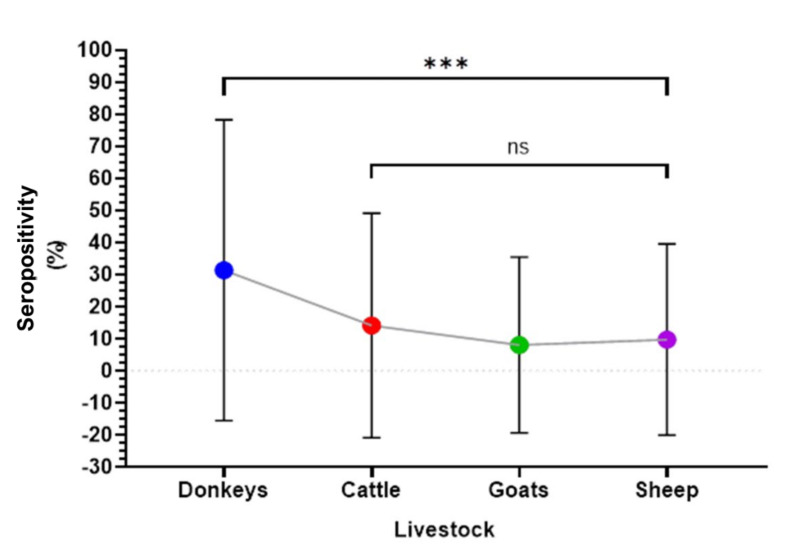
Proportion of sera containing antibodies against CCHFV in various livestock species. Seropositivity was highest in donkeys (31.4%), then cattle (14.2%), goats (9.8%), and least in sheep (8.1%). There was a significant difference in seropositivity between donkeys and the other livestock species (cattle, goats and sheep). However, there was no significant difference among cattle, goats and sheep. The error bars represent 95% confidence intervals. ***: Highly significant, ns: not significant at the 0.05 level.

There was a significant difference in the proportion of past infections among the livestock species (χ^2^ = 15.20, df =3, *p* = 0.001). Seropositivity was highest in donkeys (31.4%, 16/51) and lowest in sheep (8.1%, 24/295) (Figure 2).

#### 3.1.3. CCHFV Antibody Detection in Rodents

In total, 93 rodent sera, including 37 from Marigat and 56 from Nguruman and 54 females and 39 males, were screened for the presence of CCHFV IgG antibodies. Overall, seropositivity of 6.5% (6/93) was found, which is higher but non-significantly different in Nguruman (5/56, 8.9%) compared to Marigat (1/37, 2.7%) (95% CI, 3.5 (0.4–42.6), *p*= 0.23), as well as in females (5/54, 9.3%) compared to males (1/39, 2.5%) (95% CI, 3.9 (0.5–46.7), *p*= 0.19) (Table 6). Female rodents were 3.9 times more likely to be positive for antibodies against CCHFV compared to males. The risk of CCHFV seropositivity did not vary by age group (RR = 1.0, df 1, *p* = 0.9705) (Table 7).

**Figure 3:**
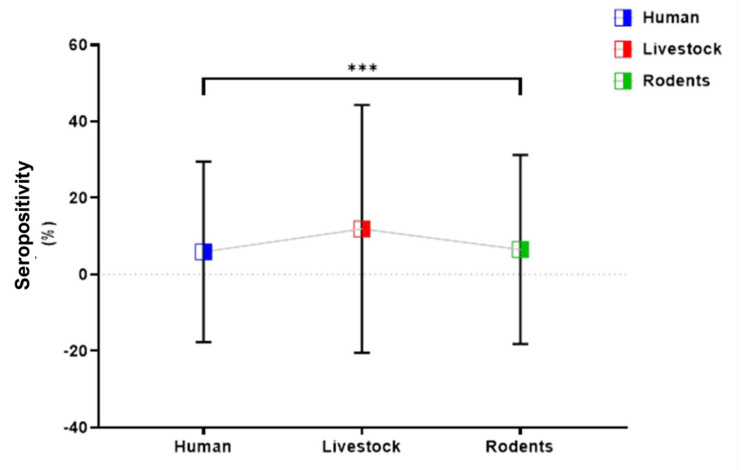
Overall seropositivity of CCHFV in humans, livestock, and rodents. The seropositivity was significantly higher in livestock (11.9%) compared to human and rodents. There was no significant difference between humans (5.9%) and rodents (6.5%). The error bars represent 95% confidence intervals. ***: Highly significant at the 0.05 level.

Comparison of the CCHFV seropositivity rate disaggregated by animal groups, such as livestock (11.9%), rodents (6.5%), and humans (5.9%), revealed a significant difference (*p* = 0.001) (Figure 3). A significantly higher probability of CCHFV infection in livestock than in humans was found (OR = 95% CI; 2.2 (1.4–3.3), RR = 1.7 (1.3–2.5), df = 1, *p* = 0.0003). However, rates did not differ between humans and rodents (OR = 95% CI; 1.1 (0.5–2.6), RR = 1.0 (0.9–1.3), df = 1, *p* = 0.83) and not between livestock and rodents (OR = 95% CI; 2.0 (0.9–4.2), RR = 1.0 (0.9–1.1), df = 1, *p* = 0.12).

### 3.2. Detection of CCHFV in Livestock, Rodents and Humans

A total of 493 and 480 individual human and rodent samples, respectively, as well as 280 pooled livestock samples were screened for CCHFV by RT-PCR. Of these, four sheep pools (three originating from Marigat and one from Nguruman) and four rodent samples (originating from Marigat) contained CCHFV RNA. CCHFV was detected in one *Rattus rattus* and three Mus musculus (Figure 1). PCR amplicons were obtained for three human and five cattle samples, but sequencing was unsuccessful. Attempts to obtain more sequence information from the positive samples using NGS were not successful.

**Figure 4:**
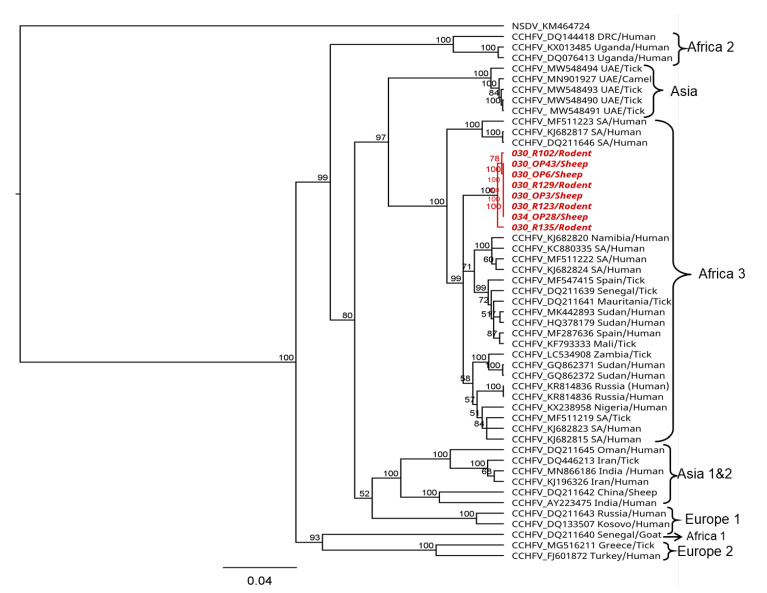
Phylogenetic relationship of CCHFV sequences detected in sheep and rodents from Kenya. Maximum likelihood (ML) phylogenetic analysis was based on a 536-nucleotide fragment of the nucleoprotein (S segment). Sequences were aligned using MAFFT and phylogenetic analyses and were inferred using PhyML v. 2.2.4 with the GTR substitution model employing 1000 bootstrap replicates. Sequences from this study are shown in red. Sequence accession number, country of origin, and host or vector species are indicated.

The partial CCHFV S segment sequences of 536 nucleotides generated both from sheep and rodents showed pairwise nucleotide identities of up to 99% among each other and of 96–98% to strains in the CCHFV Africa 3 lineage. Phylogenetic analyses showed that the sequences from this study formed a sub-clade within the Africa lineage 3 (Figure 4).

## 4. Discussion

The data of this study show CCHFV circulation among humans, livestock, and peri-domestic rodents from two semi-arid ecologies within the Kenyan Rift Valley. Antibodies against CCHFV were detected in donkeys (31.4%), cattle (14.1%), sheep (9.8%), goats (8.1%), rodents (6.5%), and humans (5.9%). In addition, CCHFV RNA was detected in sheep (1.4%) and rodents (0.83%). The observations are consistent with previous detection of virus antibodies in febrile patients [15,16,50] and cattle [29,32] as well as wildlife [32]. Furthermore, the detection of CCHFV RNA in sheep, despite the short and low viremia, was consistent with CCHF infection in livestock [29,30,31,32,33]—an indication of active circulation at the sites. However, the detection of CCHFV RNA in peri-domestic rodents constitutes a novel finding, potentially implicating them in CCHFV epidemiology in the country. Taken together, these findings including the widespread detection of ticks [30,31] suggest that the virus is endemic in some regions of Kenya.

CCHFV infection in humans is lethal, with high fatality rates of up to 40% (WHO) during outbreaks depending on the infecting strain. Here, we detected IgM antibodies against CCHFV in nine cases of fevers of unknown origin (1.8%, 9/493), indicating evidence of recent or acute CCHFV infection, which may be the cause of febrile illness in these patients. However, the contribution of other aetiologies cannot be discounted, as fever is a common symptom of other local disease conditions, often with similarity in clinical presentation to those of arboviral pathogens [3,65]. The presence of antibodies against CCHFV in humans was not affected by gender, but by location and age, being higher in Marigat than Nguruman (8/9 88.9% (95% CI 54.3–100) and 1/9, 11.1% (95% CI (1–45.6), respectively), and in the age group ≥18 years (6/9, 66.7% (95% CI 35.1–88.3)). Geographic differences in risk profile could also be attributed to other factors, such as the abundance of competent tick species, exposure of humans to tick bites or degree of contact with animals. The higher presence of IgM and IgG antibodies in older patients was associated with the type of occupation and long-term involvement in activities that require frequent contact with livestock. Our data show that humans in contact with animals were more likely to be CCHFV-seropositive at both study sites. Interestingly, there was a strong positive association between CCHFV IgM-positive patients and retro-orbital pain, suggesting that this symptom could be a marker for CCHFV infection in the study area. Nevertheless, detailed socio-economic studies are needed to quantify the burden of CCHFV infection in humans.

Here, an overall CCHFV seropositivity of 14.19% was found in cattle, while a previous study found rates of 28% in cattle in Narok and Laikipia counties [29]. Seroprevalence was particularly higher in donkeys (31%), while the lowest rates were observed among small ruminants (sheep and goats). These data highlight the potential different virus exposure rates depending on the feeding preferences of tick species [29]. The likelihood of coming into contact with an infected tick and becoming infected with CCHFV was shown to increase with age [14,27,66] and is consistent with our findings of increased CCHFV seropositivity in older livestock, e.g., 10.2% in one-year-old animals, 11.1% in two-year-old animals, and 16.5% in three-year-old animals. This might also be true for the higher seropositivity rates detected in donkeys, which were older compared to other livestock species. CCHFV seropositivity in livestock may reach up to 80% in certain livestock species and regions [23]. For example, higher CCHFV seropositivity rates were found in Uganda (75.0%) [25], Senegal (57.1%) [27], Mali (13–95%) [67], Sudan (21% and 19.1%) [68], Mauritania (15–80%) [66,69], and South Africa (12.7%) [68,70], but lower seroprevalence rates of 3.13% were observed in Egypt [71]. The differences in CCHFV seroprevalence rates in livestock could be due to the implementation of tick control programs using acaricides and dips, or due to changes in tick species infesting different livestock species. For example, Ogola et al. (2022) found differential infestation of *Hyalomma marginatum*, the principal vector and reservoir of CCHFV, on sheep but not on other livestock species in the same study area [49].

This study presents the first known detection of CCHFV RNA in rodents in Kenya. CCHFV sequences were found in house rats (*Rattus rattus*) and house mice (*Mus musculus*), both species known to inhabit human dwellings. *Rattus rattus* (house rat) hosts a wide variety of harmful internal and external parasites, like fleas and tick larvae, that live on these rats and harbor disease-causing microorganisms that can infect humans, livestock, and other animals resulting in serious diseases [35,36]. *Mus musculus* lives as a human commensal in close association with humans, in houses and granaries [72,73,74]. The close interaction of these rodents with humans and livestock pastures may facilitate CCHFV transmission, e.g., by immature ticks that are mainly infesting rodents. However, in the absence of any evidence that CCHFV is a persistent infection and/or present in rodent excreta, it is unlikely that the virus can be transmitted via the same route as arenaviruses or hantaviruses. Further evidence of excretion will be required to justify such a form of transmission.

The role of rodents in CCHFV transmission is not well known, although various studies have confirmed the presence of antibodies in rodents from Africa (South Africa, Zimbabwe, Senegal, Mauritania, and Central Africa Republic), Asia (Pakistan, Iraq, and Iran) and Europe (Hungary) [7,17,23,26,38]. In this study, we report an overall seropositivity of 6.5% (6/93, 95% CI 2.7–13.6). Sera from five *Mastomys natalensis* from Nguruman and one *Rattus rattus* from Marigat contained antibodies against CCHFV. The presence of antibodies in multimammate mice (*Mastomys* spp.) has also been reported in Mauritania (27%) and South Africa/Zimbabwe (0.3%) [26,39]. Nonetheless, the small sample size for seropositivity may not present conclusive results, and further studies with larger sample sizes and robust optimized serological assays are recommended to ascertain the true CCHFV seroprevalence in rodents. Generally, the presented data are inconclusive in confirming rodents as reservoir hosts for CCHFV, although it does suggest that they could facilitate virus amplification. Further investigation is necessary to determine whether viremia could persist, thereby supporting virus spread.

The phylogenetic analysis of the partial CCHFV nucleoprotein sequences detected in this study revealed up to 99% nucleotide identity among the sequences and 96–98% nucleotide identity among strains in the African lineage 3 that have been reported to cause disease in humans [75,76,77,78,79]. This could confirm the potential risk of the virus in circulation to cause disease in the human population, although there is a need for generating whole genome sequences to allow for detailed characterization and fine-scale inferences on the genetic diversity of circulating strains in the area. In this study, attempts to generate more genome information from the CCHFV-positive samples were not successful but would be necessary for a comprehensive phylogenetic analysis and to shed light on the genetic diversity of circulating strains in the area.

Finally, the low human and livestock seropositivity reported in this study compared to what has been previously reported in other parts of Kenya, as well as in neighboring countries, can only be attributed to well-organized and managed tick control measures practiced in the areas. However, the findings confirm the possibility of CCHFV’s contribution to febrile illnesses. Although various seroprevalence studies have reported the detection of CCHFV IgM antibodies in febrile patients in different parts of Kenya [15,16,50], the true burden of CCHFV infection and its overall contribution to febrile cases cannot be ascertained. This is because of the lack of CCHFV surveillance systems in the country and routine diagnostics in the healthcare system. Considering our findings and previous reports, it is imperative to establish robust diagnostic techniques for routine diagnosis in healthcare facilities and to equip healthcare professionals with essential knowledge on the diagnosis and management of CCHFV. This will ensure early detection and prevention through the implementation of appropriate control measures. Furthermore, the scope of our study was limited to only two counties in Kenya. Consequently, the data only provide a basis for further studies and recommend nationwide one health surveillance to determine the true burden of CCHFV, which can comprehensively inform the healthcare system.

## 5. Conclusions

Overall, the data of our study provide evidence for active circulation of CCHFV in Kenya shown via the detection of viral RNA in sheep and rodents, the presence of IgM antibodies against CCHFV in febrile patients, and the presence of IgG antibodies against CCHFV in donkeys, cattle, sheep, and goats. The presence of IgM antibodies in febrile patients suggests the possibility of the contribution of CCHFV to undiagnosed febrile cases. Together, our findings emphasize the importance of one health active surveillance in vectors, livestock, humans, and rodents to understand the transmission and circulation of CCHFV. So far, rodents and shrews that live in close proximity to humans have been mostly neglected in disease surveillance, despite the potential risk of transmitting disease.

## Figures and Tables

**Table 1:** Influence of demographics on CCHFV seropositivity among febrile patients.

Parameter	Category	N (%)	Contact with Animals n (%)	IgG Results	χ^2^, df	*p* Value
Location	Marigat	323 (65.5)	289 (89.5)	22 (6.8)	1.46, 1	0.23
	Nguruman	170 (34.5)	138 (81.2)	7 (4.1)		
Gender	Female	295 (59.8)	255 (86.4)	13 (4.4)	2.89, 1	0.09
	Male	198 (40.2)	172 (86.9)	16 (8.1)		
Age	5–10	49 (9.9)	41 (83.7)	3 (6.1)	1.53, 2	0.47
	10 ≤ 18	114 (23.1)	98 (60.1)	4 (3.5)		
	>18	330 (66.9)	288 (87.3)	22 (6.7)		
Occupation	Casual workers	3 (0.6)	1 (33)	0	6.82, 10	0.74
	Farmer	104 (21.1)	94 (90.4)	6 (5.8)		
	Non-Student	17 (3.4)	11 (64.7)	0		
	Nurse	2 (0.4)	1 (50)	0		
	Pastoralist	2 (0.4)	2 (100)	0		
	Farmhand	71 (14.4)	64 (90.1)	8 (11.3)		
	Businesspersons	27 (5.5)	22 (81.5)	2 (7.4)		
	Security	2 (0.4)	2 (100)	0		
	Student	169 (34.3)	147 (87)	10 (5.9)		
	Unemployed	97 (19.7)	83 (85.6)	3 (3.1)		

N: sample size, n: the total number of screened per category, χ^2^, df: Chi-square, degrees of freedom.

**Table 2:** Variation in the presence of IgM antibodies by gender, age, and location.

Parameter	Category	N	Ig M	χ^2^, df	*p*-Value
Gender	Female	13	4 (30.8)	2.890,1	0.27
	Male	16	5 (31.3)		
Age	5–10	3	2 (66.7)	1.993, 2	0.37
	10 ≤ 18	4	1 (25)		
	>18	22	6 (27.3)		
Location	Marigat	22	8 (36.4)	1.209, 1	0.27
	Nguruman	7	1 (14.3)		

N: sample size, n: the total number of screened per category, χ^2^, df: Chi-square, degrees of freedom.

**Table 3:** Correlation between the presence of CCHFV IgM antibodies versus symptoms of febrile patients.

CCHFV Antibody		Age	Gender	Joint Pain	Retro-Orbital Pain	Headache	Abdominal Pain
Results: IgM	Pearson Correlation	−0.184	0.005	−0.141	0.380	0.183	0.025
	Sig. (2-tailed)	0.339	0.979	0.467	0.042 *	0.343	0.896
	N	29	29	29	29	29	29

* Correlation is significant at the 0.05 level (2-tailed).

**Table 4:** Demographics of livestock sampled.

Livestock	N	Location		Sex		Age (yrs)		
Species		Marigat n (%)	Nguruman n (%)	Females n (%)	Males n (%)	1 n (%)	2 n (%)	3 n (%)
Cattle	310	170 (54.8)	140 (45.2)	207 (66.8)	103 (33.2)	70 (22.6)	177 (57.1)	63 (20.3)
Goats	295	165 (55.9)	130 (44.1)	219 (74.2)	76 (25.8)	75 (25.4)	171 (58)	49 (16.6)
Donkey	51	51 (100)	0	40 (78.4)	11 (21.6)	6 (11.8)	36 (70.6)	9 (17.6)
Sheep	295	165 (55.9)	130 (44.1)	209 (70.8)	86 (29.2)	64 (21.7)	182 (61.7)	49 (16.6)
Total	951	551 (57.9)	400 (42.1)	675 (71)	276 (29)			

N: sample size, n: is the total number of screened per category.

**Table 5:** Presence of CCHFV IgG antibodies in the livestock by sex and species.

Species	N	Within Population n (%)	Among the Total Population n (%)	Sex	Seropositive n (%)	χ2, df	*p*-Value	OR (95% CI)
Cattle	310	44 (14.19)	44 (4.63)	Female ⁑	32 (15.5)	0.82, 1	0.365	1.4 (0.68–2.74)
				Male	12 (11.7)			
Goats	295	24 (8.14c)	24 (2.52)	Female	14 (6.4)	3.46, 1	0.063	2.2 (0.94–5.19)
				Male ⁑	10 (13.2)			
Donkey	51	16 (31.37)	16 (1.68)	Female ⁑	13 (32.5)	0.11, 1	0.7407	1.3 (0.28–5.04)
				Male	3 (27.3)			
Sheep	295	29 (9.83)	29 (3.05)	Female	19 (9.1)	0.44, 1	0.665	1.3 (0.60–2.93)
				Male ⁑	10 (11.6)			

⁑: Reference category for the odds ratio, N: sample size, n: is the total number of screened per category, χ^2^, df: Chi-square, degrees of freedom.

**Table 6:** Information on rodent demographics in relation to CCHFV seropositivity.

Parameter	Level	N (%)	Positives (%)
Gender	Female	54 (58.1)	5 (9.3)
	Male	39 (41.9)	1 (2.5)
Age	Sub-Adult	78 (83.9)	5 (6.4)
	Adult	15 (16.1)	1 (6.7)
Location	Marigat	37 (39.8)	1 (2.7)
	Nguruman	56 (60.2)	5 (8.9)
Species	*Acomys* sp.	1 (1.1)	0
	*Aethomys* spp.	4 (4.3)	0
	*Arvicanthis* spp.	6 (6.5)	0
	*Gerbilliscus* spp.	3 (3.2)	0
	*Grammomys* spp	2 (2.2)	0
	*Graphiurus* sp.	1 (1.1)	0
	*Mastomys* spp.	48 (51.6)	5 (10.4)
	*Mus* sp.	1 (1.1)	0
	*Paraxerus* sp.	1 (1.1)	0
	*Rattus* spp.	26 (28)	1 (3.8)

**Table 7:** CCHFV seropositivity in rodents.

Parameter	Category	N (%)	Positive	χ^2^, df	*p*-Value	OR, 95%CI	RR
Gender	Female	54 (58.1)	5 (9.3)	1.682, 1	0.1947	3.9 (0.5–46.7)	1.5 (0.8–2.0)
	Male	39 (41.9)	1 (2.5)				
Age	Sub-Adult	78 (83.9)	5 (6.4)	0.001370, 1	0.9705	1.0 (0.1–12.0)	1.0 (0.5–1.2)
	Adult	15 (16.1)	1 (6.7)				
Location	Marigat	37	1 (2.7)	1.431, 1	0.2316	3.5 (0.4- 42.6)	2.5 (0.7–13.9)
	Nguruman	56	5 (8.9)				

N: sample size.

## Data Availability

The metadata supporting the results of this study are available upon request from the authors. The data are not publicly accessible due to the privacy of the research participants, especially the febrile patients and livestock farmers. The partial sequences of the S segment were deposited in GenBank under the accession numbers: OQ357265-OQ357272.
